# A MAM-targeting therapeutic peptide restores autophagy homeostasis and ameliorates atherosclerosis

**DOI:** 10.7150/thno.132357

**Published:** 2026-04-16

**Authors:** Jungmin Ha, Minjeong Ko, Yong-Beom Lim, Ho Jeong Kwon

**Affiliations:** 1 Chemical Genomics Leader Research Laboratory, Department of Biotechnology, College of Life Science and Biotechnology, Yonsei University, 50 Yonsei-ro, Seodaemun-gu, Seoul 03722, Republic of Korea.; 2 Department of Materials Science and Engineering, Yonsei University, 50 Yonsei-ro, Seodaemun-gu, Seoul 03722, Republic of Korea.

**Keywords:** Mitochondria-associated ER membranes, Protein-protein interaction, Organelle crosstalk, Atherosclerosis, Peptide therapeutics

## Abstract

**Rationale:**

Mitochondria-associated ER membranes (MAMs) are critical hubs for Ca^2+^ signaling, energy homeostasis, and autophagy. Their dysregulation contributes to lipid-driven cardiovascular diseases; however, selective and reversible strategies to modulate MAM-associated protein-protein interactions (PPIs) remain limited. This study aimed to develop a targeted peptide to disrupt the IP_3_R-GRP75-VDAC1 complex and evaluate its therapeutic efficacy in atherosclerosis.

**Methods:**

Based on structural and interface analyses of the IP_3_R-GRP75 complex, we designed cell-permeable MAM-targeting peptides. The activity of the lead candidate, Peptide 4, was assessed using proximity ligation assays, microscale thermophoresis (MST) analysis, cellular thermal shift assays, co-immunoprecipitation, live-cell Ca^2+^ imaging, and autophagy flux analyses in endothelial cells and macrophages under basal and oxidized low-density lipoprotein (oxLDL)-induced stress. The therapeutic efficacy was further evaluated in Western diet-fed ApoE^-/-^ mice.

**Results:**

Peptide 4 bound to GRP75, disrupted the IP_3_R-GRP75 interaction, and selectively attenuated ER-to-mitochondria Ca^2+^ transfer. This controlled Ca^2+^ modulation modestly reduced cellular ATP levels, activated the AMPK-TFEB axis, and restored functional autophagic flux. These effects were preserved under oxLDL-induced lipid stress. Restoration of MAM architecture closely correlated with autophagy recovery and lipid clearance, indicating its potential utility as a pharmacodynamic indicator. *In vivo*, systemic administration of Peptide 4 significantly improved serum lipid profiles, attenuated aortic plaque formation, reduced cardiac lipid deposition, and normalized MAM architecture in ApoE^-/-^ mice.

**Conclusions:**

Our findings identify peptide-mediated targeting of the IP_3_R-GRP75 interaction as a promising strategy to modulate MAM structure, activate adaptive autophagy, and alleviate atherosclerotic pathology. This study supports organelle contact site modulation as both a therapeutic mechanism and a measurable disease-responsive feature, highlighting peptide-based modulation of protein-protein interactions as a promising approach for metabolic and cardiovascular diseases.

## Introduction

Beyond serving as static structural linkages, Mitochondria-associated endoplasmic reticulum membranes (MAMs) function as dynamic signaling platforms whose remodeling reflects cellular metabolic state and stress adaptation 1, 2. MAMs are maintained by defined tethering complexes that establish and dynamically regulate the distance between the two organelles, thereby influencing Ca^2+^ signaling and metabolic communication 3. At these contact sites, Ca^2+^ is transferred from the ER to mitochondria through a multi-protein complex composed of the inositol 1,4,5-trisphosphate receptor (IP_3_R) on the ER membrane, the chaperone glucose-regulated protein 75 (GRP75/HSPA9), and the voltage-dependent anion channel 1 (VDAC1) on the mitochondrial outer membrane 4, 5. The IP_3_R-GRP75-VDAC1 axis facilitates the formation of Ca^2+^ microdomains that couple Ca^2+^ signaling to mitochondrial metabolism, ATP production, and cellular energy homeostasis 6, 7. Dysregulation of ER-mitochondrial Ca^2+^ transfer has been implicated in diverse pathological processes, positioning MAMs as critical regulatory hubs with emerging therapeutic relevance 8-10.

Pathological enhancement of MAM formation has been reported in metabolic and cardiovascular diseases, where sustained mitochondrial Ca^2+^ overload causes metabolic stress, impaired autophagy, and organelle dysfunction 11-14. In particular, aberrant expansion of MAMs in endothelial cells and macrophages exposed to oxidized low-density lipoprotein (oxLDL) has been associated with defective lipid handling, inflammatory activation, and progression of atherogenesis 15, 16.

Despite increasing recognition of MAMs as therapeutic targets, strategies to selectively modulate ER-mitochondrial contacts remain limited 17. Genetic manipulation of MAM-associated proteins often results in irreversible or pleiotropic effects, while pharmacological approaches lack spatial precision and specificity. Protein-protein interactions (PPIs) play central roles in organizing signaling complexes and organelle contact sites, yet remain challenging therapeutic targets due to their large and often shallow interfaces 18. Recent advances have highlighted peptides as promising PPI modulators, owing to their ability to engage extended interaction surfaces with high specificity and tunability 19-21. Accordingly, peptide-based strategies have emerged as effective tools for selectively disrupting or stabilizing disease-relevant PPIs that are difficult to target with conventional small molecules 22, 23. Consistent with this concept, previous studies have demonstrated that short peptides derived from IP_3_R can selectively disrupt specific protein-protein interactions at mitochondria-associated ER membranes, thereby modulating ER-to-mitochondria Ca^2+^ signaling 24. However, whether direct peptide-based interference with the IP_3_R-GRP75 interface can selectively modulate ER-mitochondrial Ca^2+^ signaling without perturbing organelle integrity remains unclear.

Recent preclinical work, including our previous study, has demonstrated that pharmacological modulation of MAM-associated pathways confers protective effects *in vivo* in models of atherosclerosis, providing a rationale for targeting MAMs as a therapeutic strategy 16. However, it remains unsolved whether modulation of this specific PPI can serve not only as a therapeutic intervention but also as a measurable indicator of MAM functional state under disease conditions.

In this study, we report, for the first time, the identification and functional characterization of a MAM-targeting peptide that selectively modulates ER-mitochondrial contacts by interfering with the IP_3_R-GRP75 interaction. We demonstrate that this peptide rewires ER-mitochondrial Ca^2+^ signaling without globally disrupting ER Ca^2+^ stores, activates an AMPK-TFEB-dependent autophagy program, and restores lipid homeostasis under oxLDL-induced pathological conditions. Furthermore, we provide *in vivo* evidence that peptide-mediated modulation of MAM architecture alleviates atherosclerotic pathology. Together, these findings establish peptide-based targeting of PPIs at organelle contact sites as a viable and tunable therapeutic strategy for metabolic and cardiovascular diseases, while highlighting MAM architecture and function as disease-responsive cellular features.

## Materials and Methods

### Peptide design

Candidate peptide sequences were designed based on the conserved chaperone-substrate recognition mechanism of GRP75, a member of the Hsp70 family whose substrate-binding domain (SBD) preferentially binds to short hydrophobic peptide motifs. Because GRP75-mediated interactions at mitochondria-associated ER membranes are likely governed by this substrate-recognition logic, our design strategy focused on sequences derived either from the GRP75 SBD itself or from client protein regions predicted to resemble canonical Hsp70 substrate motifs. In particular, attention was given to the IP_3_R transmembrane domain (TMD), which contains hydrophobic sequences exposed at mitochondria-associated ER membranes and thus represents a plausible interaction interface for GRP75 binding.

Peptide 1 was derived from a region within the GRP75 SBD predicted to participate in protein-protein interactions, selected based on conserved structural and functional features of the Hsp70 family substrate-recognition domain. To examine whether spatial flexibility between the targeting sequence and the cell-penetrating peptide (CPP) influences activity, Peptide 2 was generated by introducing a glycine-rich linker (GGG) between the CPP and the GRP75-derived sequence 25. Peptide 3 was selected from a distinct region of the GRP75 SBD predicted to adopt an α-helical conformation, allowing evaluation of whether structured secondary elements commonly involved in protein-protein interactions could enhance GRP75 engagement 26. Peptide 4 was designed based on a sequence derived from the IP_3_R TMD. This region exhibits similarity to conserved hydrophobic peptide motifs recognized by Hsp70 family members, including bacterial DnaK and mammalian GRP75/HSPA9, whose substrate-binding domains engage short peptide sequences through an evolutionarily conserved recognition mechanism 27-29. This rationale supported the selection of the IP_3_R-derived sequence as a potential competitive modulator of the IP_3_R-GRP75 interaction.

All peptides were conjugated to the Hph-1 cell-penetrating peptide to facilitate intracellular delivery. Hph-1 is a protein transduction domain that enables efficient intracellular delivery of peptides and proteins in various cell types, both *in vitro* and *in vivo* 30-32. CPP-mediated uptake has been reported to occur through multiple mechanisms, including endocytosis and direct membrane translocation 33, 34. For sequence-specificity assessment, a mutant control peptide was generated by substituting key residues within the predicted interaction interface with alanine or non-interacting residues, while preserving overall peptide length and charge. Detailed peptide sequences, lengths, molecular weights, and physicochemical properties are provided in Figure S1B.

### Structural modeling and visualization

Structural modeling of the IP_3_R-GRP75-VDAC1 complex was performed using experimentally determined and predicted protein structures. The structures of IP_3_R (PDB ID: 6DRC) and VDAC1 (PDB ID: 6G6U) were obtained from the Protein Data Bank. The predicted structure of GRP75 was obtained from the AlphaFold Protein Structure Database. Structure-based modeling was used to support the interpretation and visualization of protein-protein interaction interfaces. Following functional identification of Peptide 4, the IP_3_R-GRP75 interaction interface was analyzed using the ClusPro protein-protein docking server (version 2.0) to predict putative binding regions and to aid structural visualization of the interaction 35. The resulting models were used to visualize the predicted interaction interface. Molecular visualizations were generated using PyMOL (Schrödinger, LLC, New York, NY). For Figure S2C, multimeric structural models of GRP75 in complex with Peptide 4 or its mutant were generated using AlphaFold-Multimer. These predicted structures were visualized using Discovery Studio Client 2024 software (BIOVIA, San Diego, CA).

### Peptide synthesis and purification

All peptides used in this study were custom-synthesized by HLB PEP (Gwangju, South Korea) using standard solid-phase peptide synthesis. Peptide purity (> 95%) was confirmed by reversed-phase high-performance liquid chromatography (RP-HPLC), and molecular weight was verified by MALDI-TOF mass spectrometry, as provided in the manufacturer's certificate of analysis. Lyophilized peptides were supplied as trifluoroacetate (TFA) salts and appeared as white amorphous powders. Peptides were reconstituted in sterile water or phosphate-buffered saline (PBS), as appropriate, aliquoted, and stored at -20 °C. Working solutions were freshly prepared by dilution in complete cell culture medium immediately prior to each experiment.

### Microscale thermophoresis (MST) assay

Recombinant His-tagged GRP75 protein (NBC1-18380, Novus Biologicals) was fluorescently labeled using the RED-tris-NTA 2nd Generation labeling kit (MO-L018, NanoTemper Technologies, Munich, Germany) according to the manufacturer's instructions. Binding assays were carried out using a Monolith NT.115pico (NanoTemper Technologies). Labeled GRP75 was diluted to a final concentration of 20 nM in MST buffer (PBS supplemented with 0.05% Tween-20) and incubated with serial dilutions of peptide samples. The mixtures were loaded into premium-coated capillaries (MO-K025, NanoTemper Technologies), and thermophoresis measurements were recorded. Dissociation constants (K_d_) were calculated using MO.Affinity Analysis software (NanoTemper Technologies).

### Cell culture and treatment

Human umbilical vein endothelial cells (HUVECs, passages 4-9; Lifeline Cell Technology, Frederick, MD) were cultured in EBM-2 (Lonza, Basel, Switzerland) supplemented with the manufacturer-provided growth factors. HeLa cells (Korean Cell Bank, Seoul, South Korea) and murine macrophage cell line RAW264.7 (Korean Cell Bank, Seoul, South Korea) were cultured in DMEM containing 10% FBS and 1% antibiotics. Cells were maintained at 37 °C in a humidified incubator with 5% CO_2_ and routinely tested and confirmed to be free of mycoplasma contamination.

For peptide treatment, cells were seeded at appropriate densities and allowed to adhere overnight prior to experiments. Peptides were freshly prepared and diluted in complete culture medium to the indicated concentrations. Cells were treated with peptides for the specified durations for each assay, while vehicle-treated cells served as controls.

For oxLDL-induced foam cell and lipid accumulation assays, cells were incubated with oxidized low-density lipoprotein (oxLDL; L34357, Invitrogen, Thermo Fisher Scientific, Waltham, MA) at the indicated concentration and duration. Where indicated, peptides were added concurrently with oxLDL or after oxLDL pre-treatment, as specified in the figure legends.

For pharmacological modulation experiments, cells were pretreated with inhibitors such as chloroquine (CQ; C6628, Sigma-Aldrich, St. Louis, MO), Compound C (P5499, Sigma-Aldrich), or STO-609 (HY-19805, MedChemExpress, Princeton, NJ) for the indicated durations, prior to or together with peptide treatment, as indicated in the corresponding figure legends.

### Transfection

Cells were transfected with the SPLICS_L_ vector (ER-Long β11 with OMM-GFP1-10, kindly provided by Dr. D.H. Cho (Kyungpook National Univ., Korea)) using Lipofectamine 3000 reagent (L3000015, Invitrogen) for 24 h according to the manufacturer's instructions. For autophagy flux analysis, cells were transfected with the mRFP-GFP-LC3 plasmid using Lipofectamine LTX transfection reagent (15338100, Invitrogen) for 24 h according to the manufacturer's instructions. For gene knockdown experiments, cells were transfected with the 50 nM siATG7 (L-020112-00-0005, Dharmacon, Lafayette, CO) or 50 nM siGRP75 (L-004750-00-0005, Dharmacon) using Lipofectamine RNAiMAX transfection reagent (13778150, Invitrogen) for 24-48 h according to the manufacturer's instructions.

### SPLICS-based mitochondria-ER contact assay

Mitochondria-ER contact sites were analyzed using the SPLICS (split-GFP-based contact site sensor)-probe system according to established protocols 36, 37. Cells were transfected with the SPLICS_L_ construct and incubated for 24 h prior to analysis. To enhance ER-mitochondria coupling, cells were pretreated with ethanol (10 mM) for 24 h, as previously reported 12, followed by treatment with the indicated peptides. Images were acquired across multiple Z-planes using an LSM980 confocal microscope (Zeiss, Oberkochen, Germany). Mitochondria-ER contacts were quantified as the GFP-positive SPLICS signal area normalized to the total cell area (%) using ImageJ software (ver. 1.53).

### Calcium analysis

Cells were seeded on 8-well glass-bottom slides and loaded with compartment-specific Ca^2+^ indicators: 2 μM Rhod-2-AM (mitochondrial Ca^2+^, R1244, Invitrogen), 2 μM Fluo-4-AM (cytosolic Ca^2+^, F14201, Invitrogen), or 2 μM Mag-Fluo-4-AM (ER Ca^2+^, M14206, Invitrogen) for 30 min at 37 °C. To validate mitochondrial localization of Rhod-2-AM, cells were co-stained with 100 nM MitoTracker (M7512, Invitrogen). Following dye loading, cells were incubated in Ca^2+^-free Krebs-Ringer-HEPES buffer (pH 7.4) for an additional 30 min to allow complete de-esterification and to minimize extracellular Ca^2+^ influx.

To monitor mitochondrial Ca^2+^ dynamics, time-lapse images were acquired at 5-s intervals for 3 min using a confocal microscope operated in time-series mode. Fluorescence intensities were quantified per cell using ImageJ and normalized to baseline values. Data are presented as F/F₀, where F represents fluorescence intensity at each time point, and F₀ denotes the mean fluorescence intensity averaged over the initial baseline frames. Normalized fluorescence traces were plotted as a function of time. Images were acquired using an LSM980 confocal microscope (Zeiss).

### Proximity Ligation Assay (PLA)

HUVECs were cultured on chamber slides and treated with each peptide at 50 μM for 1 h. Following treatment, PLA was performed using a Duolink *in situ* red kit (Sigma-Aldrich, DUO92101) according to the manufacturer's instructions. The following primary antibodies were used: IP_3_R-I/II/III (sc-377518, Santa Cruz Biotechnology, Dallas, TX), VDAC1 (ab34726, Abcam, Cambridge, UK), Rab7 (9367, Cell Signaling Technology, Danvers, MA), Rab7 (sc-376362, Santa Cruz Biotechnology), Protrudin (12680-1-AP, Proteintech), Mfn2/Mitofusin2 (sc-515647, Santa Cruz Biotechnology), ORAI1 (NBP1-85463, Novus Biologicals, Centennial, CO) and Stim1 (sc-166840, Santa Cruz Biotechnology). Images were captured using an LSM980 confocal microscope (Zeiss).

### Cellular Thermal Shift Assay (CETSA)

Cell suspensions at a concentration of 3 × 10^7^ cells/15 mL were treated with Peptide 4 at 37 °C for 1 h in a CO_2_ incubator with gentle mixing. After centrifugation, the cell pellet was washed with PBS and resuspended in 1 mL of PBS containing protease inhibitors. Samples were aliquoted into PCR tubes (~100 μL/tube) and heated to 40-60 °C for 3 min, then cooled to 25 °C for an additional 3 min in a thermal cycler. The samples were then centrifuged, and the pellets were resuspended in PBS containing 0.4% NP-40 and protease inhibitors. After two freeze-thaw cycles in liquid nitrogen, lysates were centrifuged at 20,000 g for 20 min at 4 °C. The supernatants containing soluble proteins were collected and analyzed by immunoblotting.

### Co-immunoprecipitation

HUVECs treated with or without Peptide 4 were harvested and lysed using ice-cold IP lysis buffer (150 mM NaCl, 50 mM Tris-HCl (pH 7.5), 2 mM EDTA, 1% NP-40, 10% glycerol, and protease inhibitor cocktail) for 30 min. The lysate was then centrifuged at 13,000 rpm for 20 min at 4 °C. For immunoprecipitation, 2 μg of primary antibodies or normal control IgG were incubated with 50 μL of Protein G magnetic beads at room temperature for 1 h. The lysates were incubated with the antibody-coated beads overnight at 4 °C. The beads were washed, and bound proteins were eluted by boiling in SDS sample buffer and analyzed by immunoblotting.

### Immunoblotting

Soluble proteins were extracted from cells using SDS lysis buffer (50 mM Tris-HCl [pH 6.8] with 10% glycerol, 2% SDS, 10 mM dithiothreitol, and 0.005% bromophenol blue). Equal amounts of protein were separated by 7.5%, 10%, or 11.5% SDS-PAGE and transferred to polyvinylidene fluoride (PVDF) membranes (BioRad, USA). Blots were then blocked and immunolabeled overnight at 4 °C with the following primary antibodies; anti-LC3B (ab48394, Abcam), anti-AMPK (2532, Cell Signaling Technology), anti-phospho-AMPK (2535, Cell Signaling Technology), anti-IP_3_R-I/II/III (sc-377518, Santa Cruz Biotechnology), anti-VDAC1 (ab34726, Abcam), anti-GRP75 (3953, Cell Signaling Technology), anti-Hsc70/HSPA8 (A2487, Abclonal, Wuhan, China), anti-β-actin (ab6276, Abcam), anti-β-tubulin (ab6046, Abcam), anti-SQSTM1/p62 (ab155686, Abcam), and anti-ATG7 (8558, Cell Signaling Technology). Immunolabeling was visualized using an enhanced chemiluminescence kit according to the manufacturer's instructions. Images were quantified using Image Lab software (Bio-Rad Laboratories).

### MTT assay

HUVECs were seeded at 3,000 cells/well in a 96-well plate and incubated overnight. Cells were then treated with Peptide 4 at the indicated concentrations for 72 h. After incubation, 3-(4,5-dimethylthiazol-2-yl)-2,5-diphenyltetrazolium bromide (MTT; 0793, VWR International, Radnor, PA) solution was added to each well at a final concentration of 0.4 mg/mL and incubated for an additional 3 h. The medium was then removed, and the resulting formazan crystals were dissolved in dimethyl sulfoxide (DMSO; D2650, Sigma-Aldrich). Absorbance was measured at 540 nm using a Victor 3 multilabel plate reader (Perkin Elmer, Waltham, MA).

### Immunocytochemistry

Cells were treated with Peptide 4, fixed with 4% formaldehyde, and permeabilized with 0.2% Triton X-100 (Junsei Chemical, 9002-93-1). After blocking with 1% BSA (Sigma-Aldrich, A5611), the cells were immunolabeled overnight at 4 °C with TFEB antibody (13372-1-AP, Proteintech). The next day, the cells were washed with PBS and treated with fluorochrome-labeled secondary antibodies diluted in 1% BSA for 1 h at room temperature. After washing three times with PBS, the cells were mounted in a DAPI-containing mounting solution. Images were captured at 400× magnification using an LSM980 confocal microscope (Zeiss). The percentage of cells showing nuclear TFEB localization was quantified using ImageJ.

### Fluorescence staining

Lipid droplets were stained with BODIPY 493/503 (Thermo Fisher Scientific, D3922), and lysosomal activity was assessed by staining with LysoTracker Red (LTR; Thermo Fisher Scientific, L7528). Nuclei were stained with Hoechst (Thermo Fisher Scientific, 62249). For assessment of lysosomal proteolytic activity, HUVECs were incubated with DQ-BSA (10 μg/mL; Thermo Fisher Scientific) in EBM-2 medium for 1 h. Cells were then washed with PBS and treated with Peptide 4 (100 μM) for the indicated duration. Images were captured at 400× magnification using an LSM980 confocal microscope (Zeiss). Fluorescence intensity was quantified using ImageJ.

### Foam cell formation

RAW264.7 macrophage cells were seeded onto 12-well plates and cultured in medium containing 50 µg/mL oxidized low-density lipoprotein (oxLDL). After 48 h of incubation, cells were fixed with 4% paraformaldehyde (Sigma-Aldrich, 252549) and stained with Oil Red O (Sigma-Aldrich, O0625) to assess intracellular lipid accumulation and foam cell formation. Cells were visualized and imaged under a light microscope.

### ATP luminescence assay

An ATPlite 1-step Luminescence Assay Kit (6016736, Revvity) was used according to the manufacturer's instructions. The cells were seeded in black 96-well plates and incubated overnight. After peptide treatment, luminescence was measured using a Victor 3 multilabel plate reader (Perkin Elmer).

### Animals, diet, and peptide administration

Six-week-old male ApoE^-/-^ mice (B6.KOR/StmSlc-Apoeshl) were purchased from Central Lab. Animal Inc. (Seoul, South Korea). Mice were housed under specific pathogen-free conditions with a 12-h light/dark cycle and provided *ad libitum* access to food and water. All animal experiments were conducted in accordance with the guidelines approved by the Institutional Animal Care and Use Committee of Yonsei University (IACUC approval number: IACUC-A-202404-1847-02). After a two-week acclimation period, mice were randomly assigned to three groups: a vehicle-treated control group, a positive control group, and a peptide-treated group. To induce hyperlipidemia and atherosclerotic phenotypes, mice were fed a Western diet (Research Diets, D12079B) throughout the experimental period. During Western diet feeding, mice received intraperitoneal injections once every two days for 8 weeks as follows: vehicle control, cryptotanshinone (CTS; 20 mg/kg) as a positive control, or Peptide 4 (50 mg/kg) as the test treatment.

### Tissue collection and aortic plaque analysis

At the end of the experimental period, mice were euthanized and perfused with phosphate-buffered saline (PBS) via the left ventricle. The heart, aorta, and blood were collected for further analysis. For *en face* analysis of atherosclerotic lesions, the aorta was dissected from the aortic root to the iliac bifurcation, longitudinally opened, and pinned to silicone plates. The tissue was fixed in 10% formaldehyde overnight, followed by staining with Oil Red O for 16 h. After washing with PBS, stained aortas were imaged using a light microscope (NS-6T, China).

### Serum lipid analysis

At the end of the experimental period, blood samples were collected at sacrifice and allowed to clot at room temperature. Serum was centrifuged and submitted to a certified external analytical service provider for biochemical analysis. Serum triglyceride and total cholesterol levels were measured using standard enzymatic assays.

### Cryosection-based lipid staining and immunofluorescence

The base of the heart containing the aortic root was embedded in optimal cutting temperature (OCT) compound, frozen at -80 °C, and serial cryosections were prepared. For lipid staining, sections were incubated with BODIPY 493/503 (Thermo Fisher Scientific) to visualize neutral lipid accumulation. For immunofluorescence staining, sections were permeabilized with 0.3% Triton X-100 and blocked with 5% BSA. Sections were then incubated overnight at 4 °C with primary antibodies against SQSTM1/p62 (Becton, Dickinson and Company, BD610833) and CD31 (Proteintech, 28083-1-AP), followed by incubation with Alexa Fluor 488- and 594-conjugated secondary antibodies (Invitrogen, A11001 and A11012) for 1 h at room temperature. Nuclei were counterstained with DAPI. Fluorescence images were acquired using an LSM980 confocal microscope (Zeiss), and signal intensity within CD31-positive regions was quantified using ImageJ.

### Proximity ligation assay in paraffin sections

For proximity ligation assay (PLA), separate aortic sinus tissues were fixed in 4% paraformaldehyde, embedded in paraffin, and sectioned. Sections were deparaffinized in xylene, rehydrated through graded ethanol, and subjected to antigen retrieval using citrate buffer (pH 6.0). PLA was performed using the NaveniFlex Tissue Red kit (NT.MR.100.RED, Navinci Diagnostics AB, Uppsala, Sweden) according to the manufacturer's instructions. Images were captured using an LSM980 confocal microscope (Zeiss).

### Statistical analysis

All data are expressed as the mean ± SEM, as determined using GraphPad Prism (ver. 9.5.1 for Windows; GraphPad Software, Inc., San Diego, CA). Quantitative data were obtained from at least three independent experiments unless otherwise noted. Statistical analyses were performed using unpaired two-tailed Student's *t*-test or one-way ANOVA with Tukey's post-hoc test and a P-value of less than 0.05 was considered statistically significant (* indicates P < 0.05, ** indicates P < 0.01, *** indicates P < 0.001, **** indicates P < 0.0001).

## Results

### Identification of a functional MAM-targeting peptide

**Figure 1:**
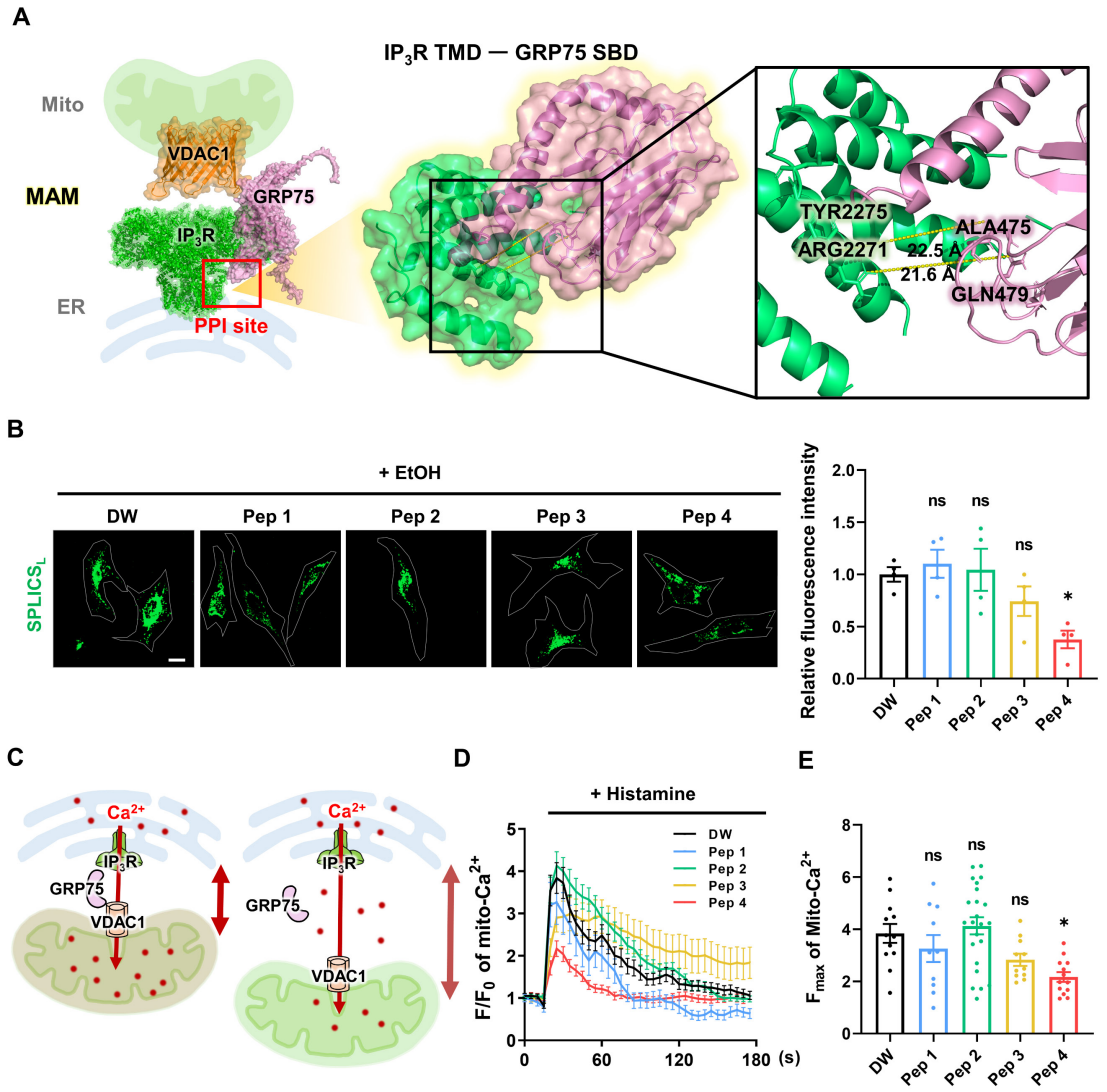
** Structure-guided identification of a functional MAM-targeting peptide.** (A) Schematic illustration and structure-based modeling of the mitochondria-associated endoplasmic reticulum membrane (MAM) complex composed of IP_3_R (green), GRP75 (pink), and VDAC1 (orange). The protein-protein interaction (PPI) site between the IP_3_R transmembrane domain (TMD) and the GRP75 substrate-binding domain (SBD), targeted for peptide design, is highlighted. An enlarged view shows the predicted IP_3_R-GRP75 interaction interface with representative inter-protein distances indicated. (B) SPLICS-based analysis of mitochondria-ER contacts in HeLa cells pretreated with ethanol (EtOH, 10 mM) to enhance ER-mitochondria coupling, followed by treatment with vehicle control (DW, distilled water) or Peptide 1-4 (Pep 1-4, 50 μM) for 1 h. Representative images and quantification of mitochondria-ER contact signals are shown. Contact frequency was quantified as the area of GFP-positive SPLICS signals relative to the total cell area (%) using ImageJ software. Scale bar, 20 μm. (C) Conceptual model illustrating the relationship between mitochondria-ER distance and mitochondrial Ca^2+^ uptake following histamine-induced ER Ca^2+^ release. Increased spatial separation between the two organelles is depicted as reduced Ca^2+^ transfer to mitochondria. (D) Live-cell time-series analysis of histamine (200 μM)-induced mitochondrial Ca^2+^ responses in HeLa cells treated with vehicle (DW) or Peptide 1-4 (50 μM) for 1 h. Representative traces showing temporal changes in mitochondrial Ca^2+^ levels, expressed as normalized fluorescence (F/F₀). (E) Quantification of maximal mitochondrial Ca^2+^ responses (F_max_) derived from the time-series measurements shown in (D). Data are presented as mean ± SEM. Statistical significance was defined as *P < 0.05; ns, not significant.

To develop a peptide capable of modulating mitochondria-ER contacts, we focused on the protein-protein interaction interface within the IP_3_R-GRP75-VDAC1 complex at mitochondria-associated ER membranes (MAMs). Based on structural information of the GRP75 substrate-binding domain and its interaction with the IP_3_R transmembrane region, a series of candidate peptides was rationally designed to target this interface (Figure 1A). This structure-guided approach aimed to selectively perturb ER-mitochondrial coupling without globally disrupting organelle integrity. The structural domains of GRP75 and IP_3_R and the residue positions of the candidate peptide sequences (Peptides 1-4) are schematically illustrated in Figure S1A.

To evaluate the functional effects of the designed peptides on MAM integrity, a SPLICS-based assay was employed to quantitatively assess mitochondria-ER contacts in living cells 36, 37. Among the tested candidates, Peptide 4 exhibited a distinct ability to modulate mitochondria-ER contact signals compared with other peptides (Figure 1B). Quantitative analysis revealed that Peptide 4 reduced SPLICS-based contact signals by 62% relative to control conditions, indicating a substantial decrease in contact frequency. This pattern indicates a partial loosening of mitochondria-ER contacts rather than complete dissociation. Given the close relationship between mitochondria-ER proximity and mitochondrial Ca^2+^ uptake, histamine-induced mitochondrial Ca^2+^ responses were next examined as a functional readout. A conceptual model illustrating reduced mitochondrial Ca^2+^ transfer upon increased mitochondria-ER distance is shown in Figure 1C.

Live-cell imaging revealed distinct temporal profiles of mitochondrial Ca^2+^ responses following peptide treatment (Figure 1D), and quantification of maximal Ca^2+^ responses further supported these observations (Figure 1E). These findings indicate that structural modulation of MAMs by Peptide 4 translates into functional attenuation of ER-to-mitochondrial Ca^2+^ transfer. In HUVECs, cell viability assessed by MTT assay revealed an IC_50_ of approximately 140 μM for Peptide 4 after 72 h treatment (Figure S1C), suggesting that the functional concentration of Peptide 4 (50 μM) is substantially below the cytotoxic range and that its observed activity is unlikely to result from nonspecific or cytotoxic effects. Efficient intracellular delivery of Peptide 4 was confirmed using FITC-labeled peptides in HUVECs (Figure S1D). Collectively, these data identify Peptide 4 as a functional MAM-targeting peptide and provide a foundation for subsequent mechanistic analyses.

### Peptide 4 selectively modulates mitochondria-ER contacts

**Figure 2:**
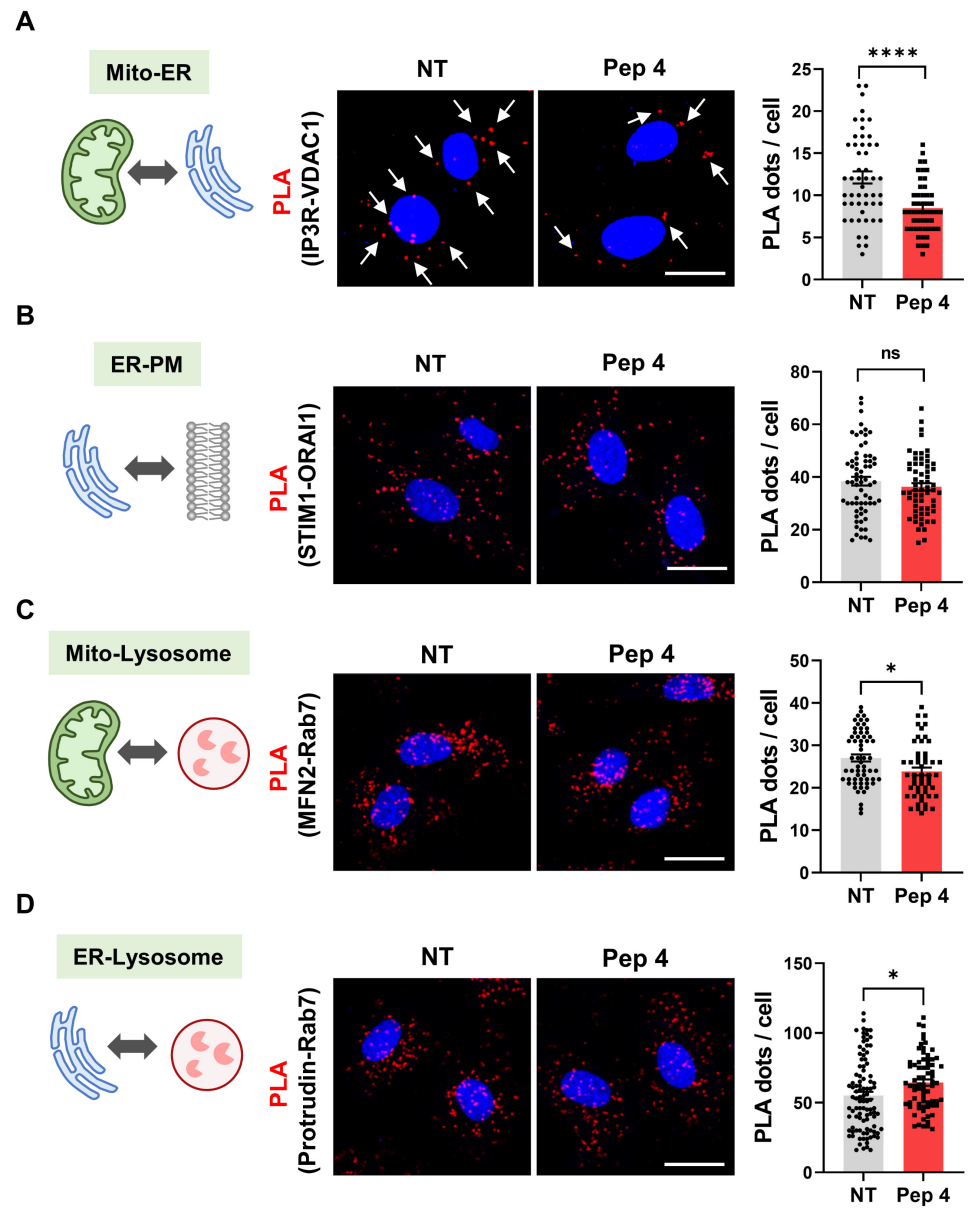
** Peptide 4 selectively modulates mitochondria-ER contacts without affecting other organelle interactions.** Human umbilical vein endothelial cells (HUVECs) were treated with Peptide 4 (Pep 4, 50 µM) for 1 h prior to fixation. (A-D) Representative proximity ligation assay (PLA) images and corresponding quantification of PLA puncta per cell showing interactions between mitochondria (Mito) and ER (A), ER and plasma membrane (PM) (B), mitochondria and lysosomes (C), and ER and lysosomes (D) under non-treated (NT) and Peptide 4-treated conditions. Nuclei were counterstained with DAPI (blue). White arrows indicate representative PLA puncta. Scale bar, 20 µm. Data are presented as mean ± SEM. Statistical significance was defined as *P < 0.05; ****P < 0.0001; ns, not significant.

To evaluate whether Peptide 4 selectively affects specific inter-organelle contact sites, proximity ligation assay (PLA) was performed to examine multiple organelle contact pairs, including mitochondria-ER (Figure 2A), ER-plasma membrane (PM) (Figure 2B), mitochondria-lysosome (Figure 2C), and ER-lysosome interactions (Figure 2D) 38-40. Among the organelle pairs examined, Peptide 4 treatment resulted in the most pronounced reduction in mitochondria-ER contact signals, as evidenced by a significant decrease in PLA puncta (Figure 2A). Quantitative analysis revealed that Peptide 4 reduced IP_3_R-VDAC1 PLA signals by 30% relative to non-treated control cells, with the strongest statistical significance among the tested organelle interactions. This selective reduction supports the notion that Peptide 4 preferentially targets MAM-associated interactions rather than nonspecifically disrupting cellular architecture.

A mutant control peptide (Pep 4 mutant), in which two conserved tyrosine residues in Peptide 4 were substituted with alanine (YY→AA), was generated to disrupt the predicted interaction interface. Quantitative analysis showed that, whereas Peptide 4 reduced mitochondria-ER contact signals by 30% relative to control conditions, the mutant peptide failed to induce a significant change in PLA puncta frequency (Figure S2A). This result underscores the requirement for precise sequence recognition to mediate the observed effects and supports a protein-protein interaction-dependent mechanism of action. In contrast, ER-plasma membrane, mitochondria-lysosome, and ER-lysosome contacts showed minimal or no significant changes upon Peptide 4 treatment (Figure 2B-D). Quantitative analysis across all contact pairs confirmed that the effect of Peptide 4 was most robust for mitochondria-ER interactions, indicating a selective modulation of mitochondria-associated ER membranes rather than a global disruption of inter-organelle contacts. To further assess whether this selective modulation is associated with direct target engagement, we performed microscale thermophoresis (MST) analysis to evaluate the binding of Peptide 4 to GRP75 (Figure S2B). Peptide 4 exhibited measurable binding affinity to GRP75 (K_d_ = 3.9 μM), whereas the mutant peptide showed substantially reduced binding affinity (K_d_ = 47 μM). These results support a sequence-dependent and preferential interaction of Peptide 4 with GRP75. In conjunction with the PLA results, these findings suggest that the observed reduction in mitochondria-ER contacts by Peptide 4 is associated with GRP75 engagement rather than nonspecific disruption of inter-organelle interactions. Having established the selectivity of Peptide 4 toward mitochondria-ER contacts, we next sought to elucidate the underlying molecular mechanism by examining its impact on the IP_3_R-GRP75 interaction and downstream ER-mitochondrial Ca^2+^ signaling.

### Peptide 4 engages the IP_3_R-GRP75 axis and reprograms ER-mitochondrial Ca^2+^ signaling

**Figure 3:**
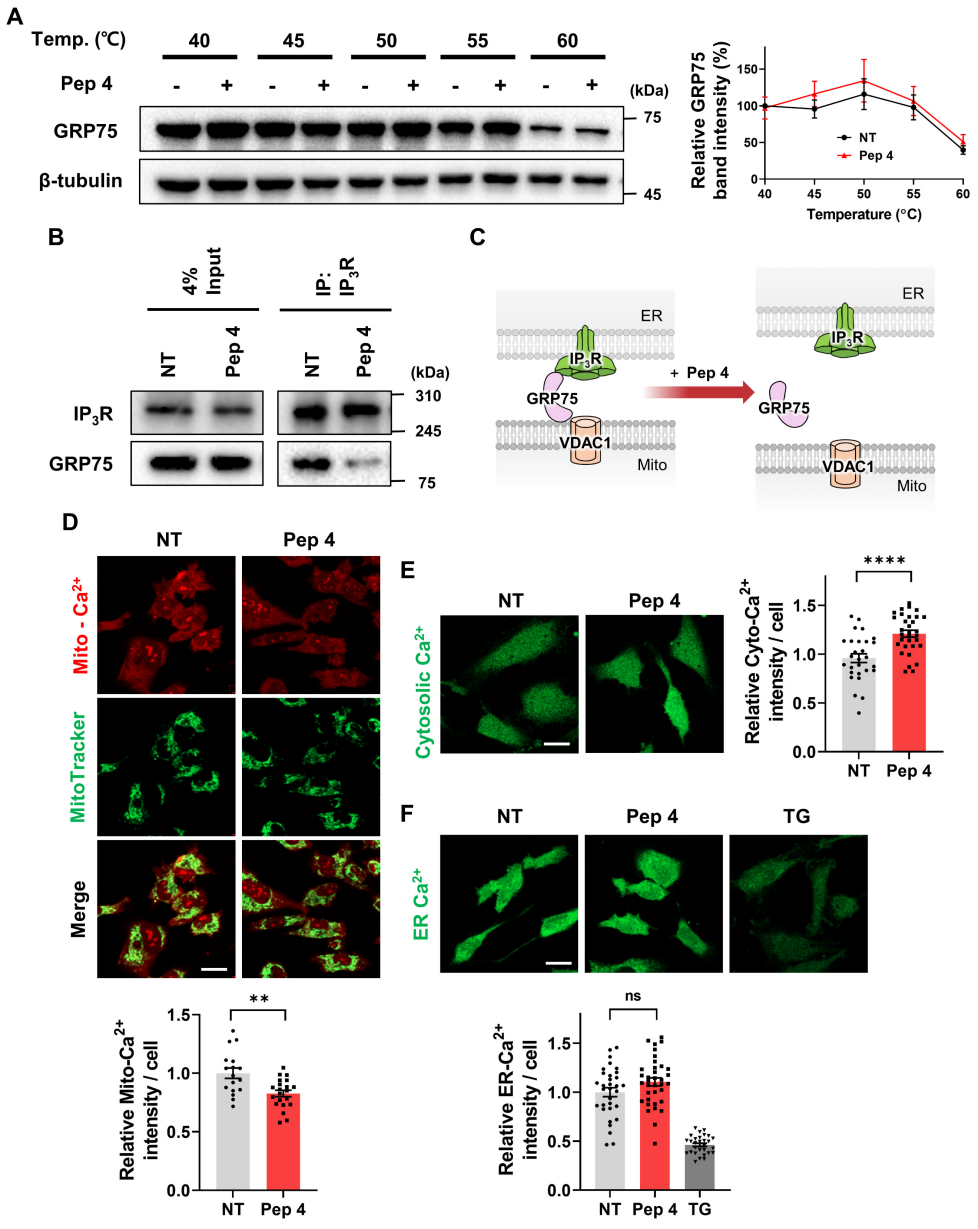
** Peptide 4 engages the IP_3_R-GRP75 axis and alters ER-mitochondrial Ca**^2+^** signaling.** Human umbilical vein endothelial cells (HUVECs) were treated with Peptide 4 or non-treated (NT) for 1 h prior to analysis. (A) Cellular thermal shift assay (CETSA) showing thermal stabilization of GRP75 following Peptide 4 treatment (100 µM). (B) Co-immunoprecipitation analysis of the IP_3_R-GRP75 interaction in NT and Peptide 4 (100 µM)-treated cells. (C) Schematic illustration depicting Peptide 4-mediated disruption of the IP_3_R-GRP75 tethering complex at mitochondria-associated ER membranes (MAMs). (D-F) Live-cell Ca^2+^ imaging analyses showing mitochondrial Ca^2+^ levels (D), cytosolic Ca^2+^ levels (E), and ER Ca^2+^ content (F) following Peptide 4 treatment (50 µM). Thapsigargin (TG, 100 nM) was used as a positive control for ER Ca^2+^ store depletion in ER Ca^2+^ measurements. Scale bar, 20 µm. Data are presented as mean ± SEM. Statistical significance was defined as **P < 0.01; ****P < 0.0001; ns, not significant.

To investigate the molecular basis underlying the selective modulation of mitochondria-ER contacts by Peptide 4, we first examined whether Peptide 4 directly engages GRP75 in human umbilical vein endothelial cells (HUVECs). Consistent with the MST-based binding analysis, cellular thermal shift assay (CETSA) demonstrated enhanced thermal stabilization of GRP75 following Peptide 4 treatment, indicating direct target engagement within cells (Figure 3A). To assess the selectivity of this interaction, CETSA was further performed on related proteins, including the Hsp70 family member HSC70/HSPA8 and MAM-associated proteins (VDAC1 and IP_3_R). Peptide 4 did not induce detectable thermal stabilization of these proteins (Figure S3A-B), suggesting that its interaction is preferentially associated with GRP75 rather than broadly affecting related chaperones or MAM components. These findings provide cellular evidence supporting preferential engagement of GRP75 by Peptide 4. Consistently, structural modeling using AlphaFold-Multimer suggested altered interaction configurations of the mutant peptide compared with Peptide 4 (Figure S2C), providing structural context for the sequence-dependent interaction observed in binding assays.

We next assessed whether Peptide 4 modulates the interaction between GRP75 and IP_3_R. Co-immunoprecipitation analysis demonstrated that Peptide 4 treatment markedly reduced the association between IP_3_R and GRP75 compared with control conditions (Figure 3B-C). Given the central role of the IP_3_R-GRP75 complex in mediating ER-to-mitochondria Ca^2+^ transfer, we next evaluated intracellular Ca^2+^ dynamics following Peptide 4 treatment. Live-cell Ca^2+^ imaging revealed a significant reduction in mitochondrial Ca^2+^ uptake upon Peptide 4 treatment (Figure 3D), accompanied by an increase in cytosolic Ca^2+^ levels (Figure 3E). This reciprocal redistribution of Ca^2+^ signals is consistent with attenuation of directed ER-to-mitochondria Ca^2+^ transfer. Notably, Peptide 4 treatment did not significantly alter ER Ca^2+^ content compared with control conditions (Figure 3F). In contrast, Thapsigargin (TG), a specific inhibitor of the sarco/endoplasmic reticulum Ca^2+^-ATPase (SERCA), was used as a positive control for ER Ca^2+^ store depletion. These results indicate that Peptide 4 selectively rewires inter-organelle Ca^2+^ signaling without inducing global ER Ca^2+^ depletion. Overall, these findings demonstrate that Peptide 4 preferentially engages GRP75, disrupts the IP_3_R-GRP75 interaction, and functionally reprograms ER-mitochondrial Ca^2+^ signaling in endothelial cells.

### Peptide 4 activates the AMPK-TFEB axis and induces functional autophagy

**Figure 4:**
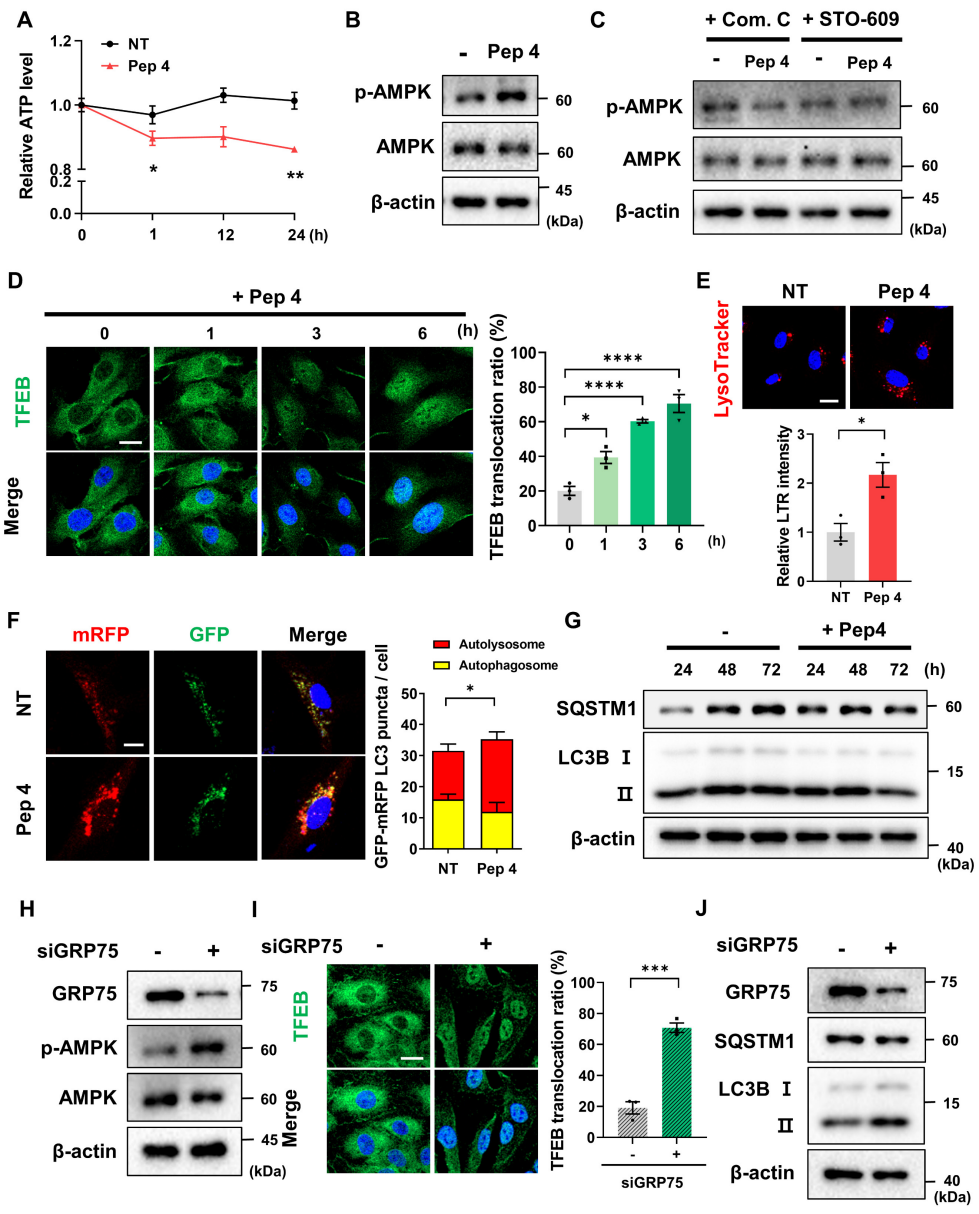
** Peptide 4 activates the AMPK-TFEB axis and induces functional autophagy.** Human umbilical vein endothelial cells (HUVECs) were treated with Peptide 4 (100 µM) or non-treated (NT) for the indicated durations prior to analysis. (A) Measurement of intracellular ATP levels following Peptide 4 treatment to assess cellular energy status. (B) Immunoblot analysis of AMPK activation, shown as phosphorylation of AMPK (p-AMPK) and total AMPK levels. (C) Immunoblot analysis of AMPK activation in cells treated with Peptide 4 in the presence of Compound C (Com. C; 20 µM) or STO-609 (10 µM). (D) Immunocytochemical analysis of TFEB subcellular localization following Peptide 4 treatment. Nuclei were counterstained with DAPI (blue). Representative images and quantification of TFEB nuclear translocation are shown. Scale bar, 20 µm. (E) Lysosomal content assessed by LysoTracker Red (LTR) staining in control (NT) and Peptide 4-treated cells (12 h). Representative images and quantification are shown. Scale bar, 20 µm. (F) Tandem fluorescent LC3 reporter analysis of autophagic flux following Peptide 4 treatment (12 h). Representative images and quantification of LC3 puncta are shown. Autophagosomes were defined as GFP⁺/mRFP⁺ puncta, and autolysosomes as mRFP-only puncta. Scale bar, 10 µm. (G) Immunoblot analysis of LC3B-I/LC3B-II conversion and SQSTM1/p62 levels following Peptide 4 treatment (100 µM) over the indicated time points (24-72 h). (H) Immunoblot analysis of AMPK activation following siRNA-mediated knockdown of GRP75 in HUVECs. (I) Immunocytochemical analysis of TFEB localization following GRP75 knockdown in HUVECs. Nuclei were counterstained with DAPI (blue). Representative images and quantification of TFEB nuclear translocation are shown. Scale bar, 20 µm. (J) Immunoblot analysis of LC3B-I/LC3B-II conversion and SQSTM1/p62 levels following GRP75 knockdown in HUVECs. Data are presented as mean ± SEM. Statistical significance was defined as *P < 0.05; **P < 0.01; ***P < 0.001; ****P < 0.0001; ns, not significant.

Given that ER-mitochondrial Ca^2+^ transfer is a key regulator of cellular energy homeostasis and autophagy signaling, we next investigated whether Peptide 4-induced alterations in Ca^2+^ dynamics activate downstream metabolic and lysosomal pathways. Measurement of intracellular ATP levels revealed a 15% reduction following Peptide 4 treatment compared with control cells (Figure 4A), indicating the induction of mild energy stress rather than cytotoxic energy collapse. Importantly, Peptide 4 treatment also increased cytosolic Ca^2+^ levels (Figure 3E), suggesting that Ca^2+^ redistribution accompanies disruption of ER-mitochondrial Ca^2+^ transfer. Consistent with this, Peptide 4 treatment activated AMP-activated protein kinase (AMPK), as evidenced by increased AMPK phosphorylation (Figure 4B). The requirement of AMPK signaling was further examined using pharmacological inhibitors. Co-treatment with either the AMPK inhibitor Compound C or the CaMKKβ inhibitor STO-609 effectively suppressed Peptide 4-induced AMPK activation (Figure 4C), indicating that AMPK activation is mediated, at least in part, through Ca^2+^-dependent CaMKKβ signaling rather than solely through changes in cellular ATP levels.

Activation of AMPK was accompanied by enhanced nuclear translocation of transcription factor EB (TFEB), a master regulator of lysosomal biogenesis and autophagy, as assessed by immunocytochemistry (Figure 4D) 41, 42. This coordinated activation of AMPK and TFEB suggests engagement of an adaptive lysosomal-metabolic program in response to altered ER-mitochondrial Ca^2+^ signaling. In line with TFEB activation, lysosomal content was markedly increased in Peptide 4-treated cells, as indicated by elevated LysoTracker staining (Figure 4E).

To determine whether these molecular changes translated into functional autophagy induction, autophagic flux was evaluated using multiple complementary approaches. Tandem fluorescent LC3 reporter (mRFP-GFP-LC3) analysis revealed a significant increase in red-only puncta following Peptide 4 treatment, indicating enhanced autophagosome maturation and lysosomal fusion (Figure 4F). Immunoblot analysis further demonstrated time-dependent conversion of LC3B-I to LC3B-II, accompanied by a decrease in SQSTM1/p62 levels over 24-72 h of Peptide 4 treatment, supporting sustained activation of autophagic flux (Figure 4G). To further examine whether these effects are mechanistically linked to the IP_3_R-GRP75 axis, we performed siRNA-mediated knockdown of GRP75 in HUVECs. GRP75 silencing resulted in increased AMPK activation (Figure 4H) and enhanced autophagy markers, including increased LC3B-I to LC3B-II conversion and decreased SQSTM1/p62 levels (Figure 4J), recapitulating the effects observed with Peptide 4 treatment. Consistent with this, immunofluorescence analysis revealed that GRP75 knockdown significantly promoted TFEB nuclear translocation (Figure 4I), supporting activation of the AMPK-TFEB axis downstream of IP_3_R-GRP75 disruption. These findings provide genetic support that disruption of the IP_3_R-GRP75 axis contributes to activation of downstream AMPK-TFEB signaling and promotes autophagic responses.

Finally, blockade of lysosomal degradation with chloroquine (CQ) abrogated Peptide 4-induced LC3B-II accumulation and p62 degradation, confirming that the observed changes reflect increased autophagic flux rather than impaired autophagosome clearance (Figure S4A). Enhanced lysosomal proteolytic activity following Peptide 4 treatment was further supported by increased DQ-BSA fluorescence (Figure S4B). Together, these results indicate that Peptide 4 enhances lysosomal function and promotes effective autophagic flux.

### Peptide 4 alleviates oxLDL-induced MAM dysregulation, restores autophagy, and reduces lipid accumulation

**Figure 5:**
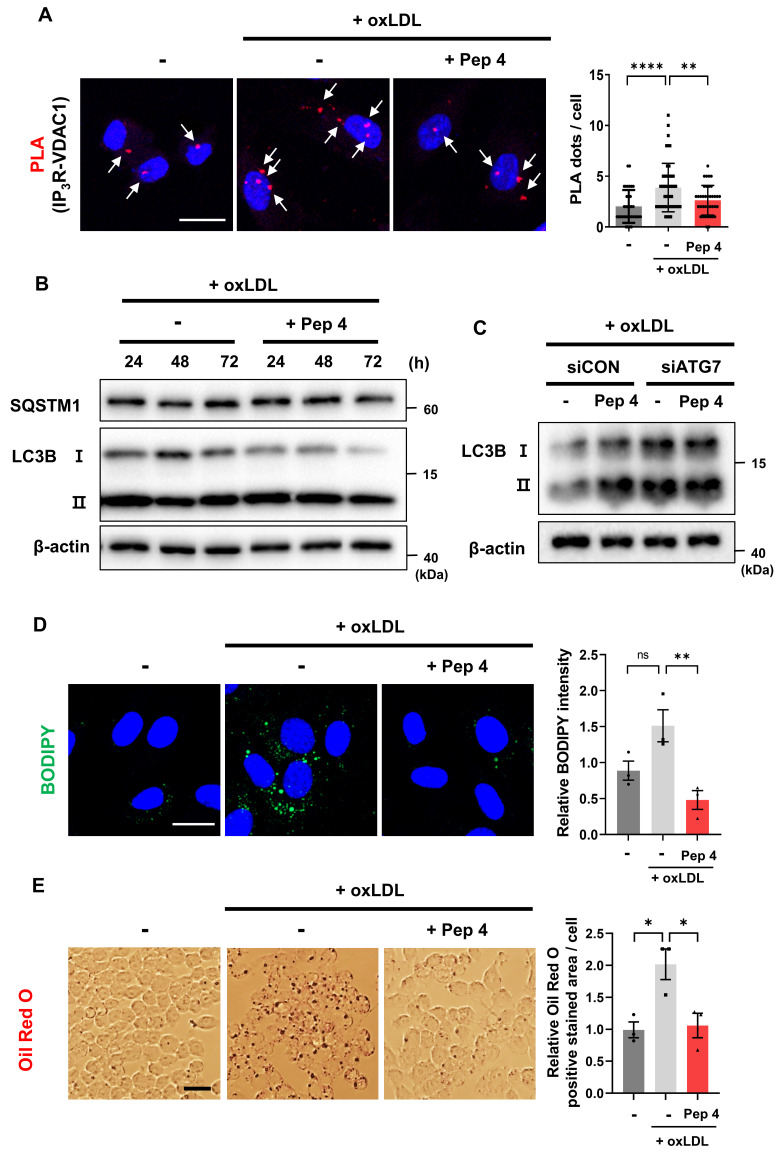
** Peptide 4 alleviates oxLDL-induced MAM dysregulation, restores autophagy, and reduces lipid accumulation *in vitro*.** Human umbilical vein endothelial cells (HUVECs) or RAW264.7 macrophages were treated with oxidized low-density lipoprotein (oxLDL, 50 µg/mL) in the absence (-) or presence (+) of Peptide 4 (Pep 4, 100 µM), as indicated. (A) PLA analysis of mitochondria-ER contacts in HUVECs treated with oxLDL alone or co-treated with Peptide 4. Representative images and quantification of PLA puncta per cell are shown. White arrows indicate representative PLA puncta. Scale bar, 20 µm. (B) Immunoblot analysis of autophagic flux markers LC3B-I/LC3B-II and SQSTM1/p62 in HUVECs treated with oxLDL in the absence or presence of Peptide 4. (C) Immunoblot analysis of LC3B-I/LC3B-II conversion in control siRNA- or ATG7 siRNA (50 nM)-transfected HUVECs treated with oxLDL and Peptide 4. (D) Intracellular lipid accumulation in HUVECs treated with oxLDL in the presence or absence of Peptide 4, visualized by BODIPY staining. Scale bar, 20 µm. (E) Lipid accumulation in RAW264.7 macrophages treated with oxLDL in the absence or presence of Peptide 4 for 48 h, assessed by Oil Red O staining. Scale bar, 20 µm. Data are presented as mean ± SEM. Statistical significance was defined as *P < 0.05; **P < 0.01; ****P < 0.0001; ns, not significant.

Given the established protective role of AMPK-TFEB-dependent autophagy against lipid overload and cellular dysfunction, we next investigated whether Peptide 4 confers functional benefits under pathophysiological conditions associated with oxidized low-density lipoprotein (oxLDL) exposure 43, 44. In human umbilical vein endothelial cells (HUVECs), oxLDL treatment markedly increased mitochondria-ER contacts, consistent with pathological MAM hyperformation 15. Co-treatment with Peptide 4 significantly attenuated oxLDL-induced increases in mitochondria-ER contacts, reducing contact frequency by 32% compared with oxLDL treatment alone (Figure 5A). Notably, this reduction reflects attenuation of elevated organelle contacts rather than suppression of basal ER-mitochondrial communication.

We next examined whether Peptide 4 restores autophagic activity impaired by oxLDL. Immunoblot analysis revealed that oxLDL treatment disrupted autophagic flux, as evidenced by reduced LC3B-I to LC3B-II conversion and accumulation of SQSTM1/p62. Co-treatment with Peptide 4 restored LC3B processing and reduced p62 levels, indicating recovery of autophagic flux under lipid overload conditions (Figure 5B). To determine whether this effect depends on an intact autophagy machinery, ATG7 was depleted using siRNA. In control siRNA-treated cells, Peptide 4 restored LC3B conversion in the presence of oxLDL, whereas this effect was abolished in ATG7-deficient cells, demonstrating that Peptide 4-induced autophagy restoration depends on canonical ATG7-mediated autophagosome formation (Figure 5C; Figure S5A).

We next assessed whether restoring autophagy with Peptide 4 reduces lipid accumulation. In HUVECs, oxLDL-induced intracellular lipid accumulation, visualized by BODIPY staining, was significantly reduced by Peptide 4 treatment (Figure 5D). Similarly, GRP75 knockdown decreased lipid accumulation (Figure S5B), supporting that modulation of the IP_3_R-GRP75 axis contributes to improved lipid handling. Consistently, lipid accumulation in oxLDL-treated RAW264.7 macrophages was also decreased upon Peptide 4 treatment, with Oil Red O-positive lipid content reduced by 47% compared with oxLDL treatment alone (Figure 5E). Collectively, these findings demonstrate that Peptide 4 mitigates oxLDL-induced pathological MAM remodeling, restores ATG7-dependent autophagic flux, and reduces lipid accumulation in both endothelial cells and macrophages, supporting its protective role under atherogenic conditions. Having demonstrated that Peptide 4 restores autophagy and alleviates lipid accumulation under oxLDL-induced pathological conditions *in vitro*, we next sought to determine whether modulation of MAM architecture by Peptide 4 confers therapeutic benefit *in vivo* using a Western diet-induced atherosclerosis mouse model.

### Peptide 4 exerts therapeutic efficacy in an atherosclerosis mouse model

**Figure 6:**
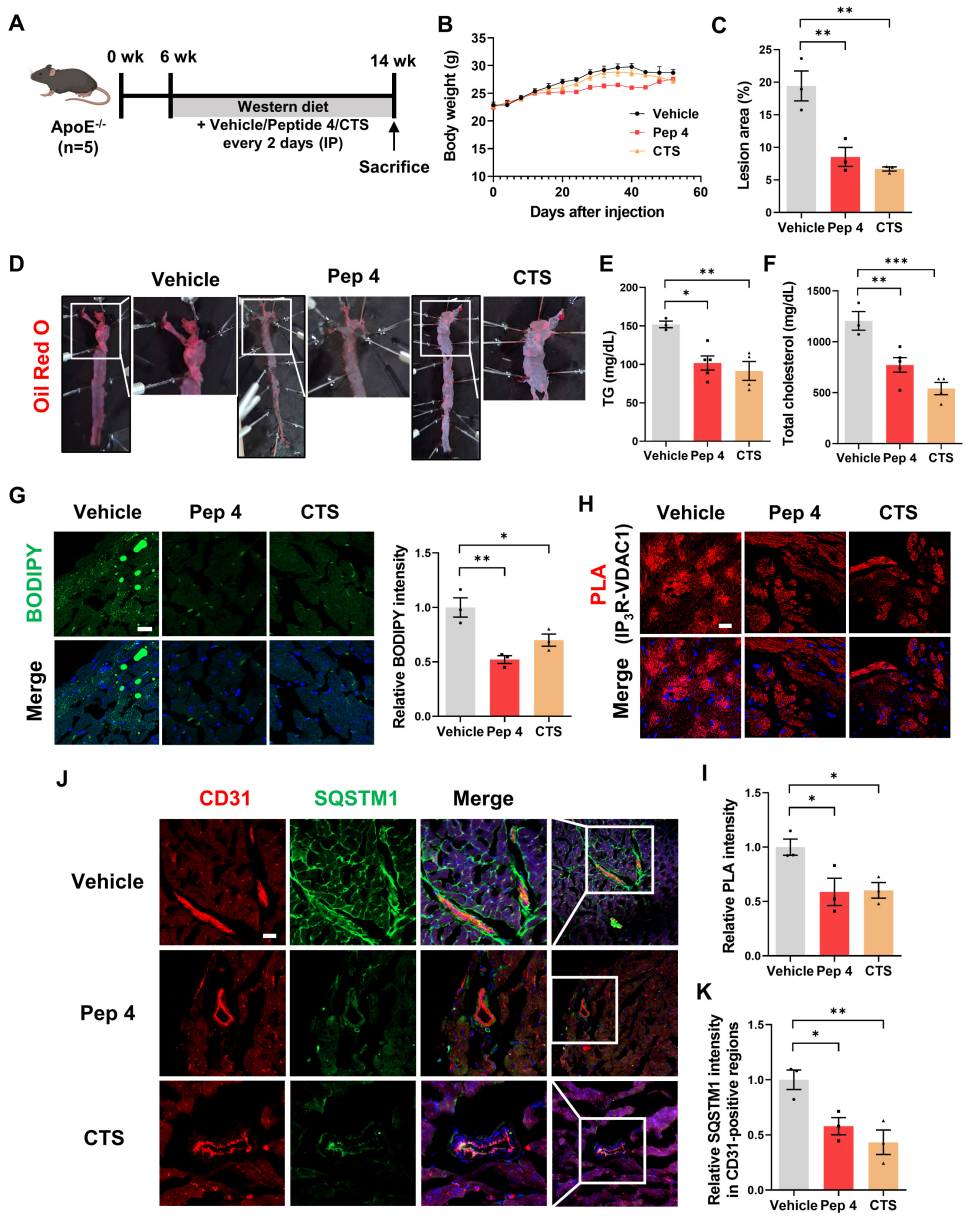
***In vivo* therapeutic efficacy of Peptide 4 in a Western diet-induced atherosclerosis model.** (A) Schematic illustration of the *in vivo* experimental timeline. ApoE^-/-^ mice were fed a Western diet and administered vehicle, Peptide 4 (50 mg/kg), or CTS (cryptotanshinone, 20 mg/kg) via intraperitoneal injection (IP) every two days for 8 weeks (n = 5 per group). (B) Body weight changes were monitored throughout the treatment period. (C) Quantification of atherosclerotic lesion area (%) shown in (D) (n = 3). (D) Representative *en face* Oil Red O staining images of whole aortas. Boxed regions indicate areas shown at higher magnification. (E-F) Serum triglyceride (TG) and total cholesterol levels were measured at the end of the experiment (n = 3-5). (G) Representative BODIPY staining images of aortic sinus sections showing lipid accumulation. Corresponding quantification is shown. Scale bar, 20 µm. (H) Proximity ligation assay (PLA) analysis of IP_3_R-VDAC1 interactions in paraffin-embedded aortic sinus sections. Representative images are shown. Scale bar, 20 µm. (I) Quantification of PLA signal intensity. (J) Immunofluorescence staining of CD31 and SQSTM1/p62 in aortic sinus sections. Representative images are shown. Scale bar, 20 µm. (K) Quantification of SQSTM1/p62 fluorescence intensity in CD31-positive regions. Data are presented as mean ± SEM. Statistical significance was defined as *P < 0.05; **P < 0.01; ***P < 0.001.

To evaluate the *in vivo* therapeutic potential of Peptide 4, its effects were examined in a Western diet-induced atherosclerosis model using ApoE^-/-^ mice. Mice were administered Peptide 4 via intraperitoneal injection every two days throughout the Western diet feeding period, according to the experimental timeline (Figure 6A). Peptide 4 treatment did not significantly affect body weight during the experimental period (Figure 6B), suggesting that repeated systemic administration was well tolerated without overt metabolic or systemic toxicity. *En face* Oil Red O staining of whole aortas revealed a marked reduction in atherosclerotic plaque burden in Peptide 4-treated mice (Figure 6D), which was further confirmed by quantitative analysis of lesion area (Figure 6C). Consistently, peptide 4 significantly improved circulating lipid profiles, as evidenced by reduced serum triglyceride and total cholesterol levels compared with vehicle-treated controls (Figure 6E-F). These findings indicate that Peptide 4 effectively suppresses diet-induced lipid deposition and plaque formation at the vascular level.

**Figure 7:**
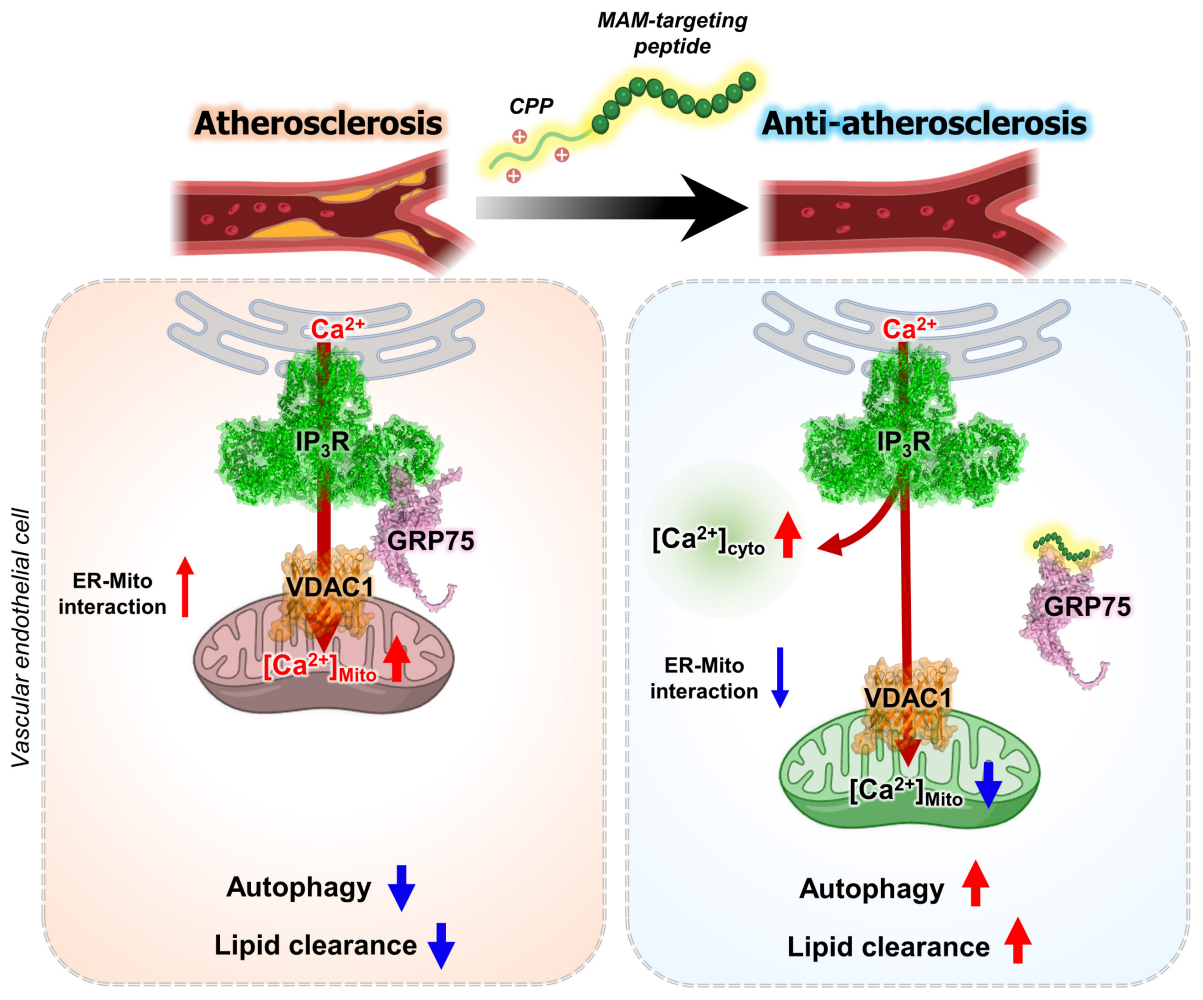
** A schematic model illustrating the therapeutic mechanism of Peptide 4 via MAM disruption in endothelial cells.** Schematic illustration showing that Peptide 4 disrupts IP_3_R-GRP75-VDAC1 tethering complex at mitochondria-associated ER membranes (MAMs) in vascular endothelial cells. Under oxidized LDL conditions, enhanced ER-mitochondria coupling increases mitochondrial Ca^2+^ transfer and impairs autophagy. Peptide 4 reduces ER-mitochondria interaction and mitochondrial Ca^2+^ uptake while increasing cytosolic Ca^2+^ levels, thereby promoting autophagy and attenuating atherosclerosis. Some elements of this figure were created using BioRender.com.

Analysis of aortic sinus sections further demonstrated that Peptide 4 significantly reduced lipid accumulation, as assessed by BODIPY staining (Figure 6G). Moreover, proximity ligation assay analysis of aortic sinus sections revealed a decrease in IP_3_R-VDAC1 interaction signals in Peptide 4-treated mice compared with controls (Figure 6H-I), providing *in vivo* evidence that Peptide 4 modulates mitochondria-ER contact sites within atherosclerotic lesions. To determine whether Peptide 4 induces autophagy activation *in vivo*, immunofluorescence analysis of SQSTM1/p62 was performed in aortic sinus sections. Notably, Peptide 4 treatment reduced p62 signal intensity in CD31-positive endothelial regions (Figure 6J-K), consistent with enhanced autophagic activity in vascular endothelial cells. Collectively, these results demonstrate that Peptide 4 exerts therapeutic efficacy against diet-induced atherosclerosis without inducing detectable toxicity, accompanied by improved lipid homeostasis and modulation of mitochondria-ER contacts* in vivo*. A schematic summary illustrating the proposed mechanism of Peptide 4, focusing on the disruption of the IP_3_R-GRP75 interaction at mitochondria-ER contact sites in vascular endothelial cells, is presented in Figure 7.

## Discussion

In this study, we show that selective modulation of a defined protein-protein interaction within mitochondria-associated ER membranes may provide a therapeutic framework for restoring autophagy homeostasis in atherosclerosis. By directly targeting the IP_3_R-GRP75 interface with a rationally designed, cell-permeable peptide, we attenuated ER-to-mitochondria Ca^2+^ transfer without disrupting global ER Ca^2+^ homeostasis or organelle integrity. This target regulation was associated with activation of the AMPK-TFEB axis, restoration of autophagic flux under lipid stress, and reduced lipid accumulation and atherosclerotic pathology *in vivo*. Importantly, these findings suggest that MAM-resident Ca^2+^ signaling represents a dynamically regulatable and disease-responsive therapeutic node, rather than a passive structural feature of organelle organization.

Accumulating evidence indicates that MAM dysregulation contributes to chronic metabolic and inflammatory diseases; however, most previously reported interventions modulate MAM function indirectly, such as through upstream stress pathways, broad Ca^2+^ channel inhibition, or genetic perturbation of tethering components 45. These approaches often lack spatial precision and may interfere with physiological Ca^2+^ dynamics required for cellular responses. In contrast, our approach was designed to act at a defined protein-protein interaction interface within MAMs. By targeting the IP_3_R-GRP75 interaction, we aimed to attenuate pathological ER-to-mitochondrial Ca^2+^ transfer while preserving Ca^2+^ signaling necessary for homeostatic responses. Recent work has demonstrated a functional association between the transmembrane-proximal region of IP_3_R and the substrate-binding domain of GRP75 in ER-mitochondrial Ca^2+^ communication 46. This observation provides independent support for our rationale in selecting this interface as the basis for designing an IP_3_R-derived peptide to modulate MAM function. Focusing on this interaction, our findings suggest that interface-level intervention can attenuate pathological MAM signaling while sparing essential physiological functions. Consistent with this view, recent studies have highlighted that mitochondrial function and stress adaptation are dynamically regulated by protein quality control and ubiquitin-dependent signaling at the organelle interfaces, underscoring the importance of precisely modulating mitochondrial regulatory networks to maintain cellular homeostasis 47.

Several peptide-based therapeutic strategies have been explored for atherosclerosis, including ApoA-I mimetic peptides such as 4F and 5A, which enhance cholesterol efflux 48, 49, Annexin A1-derived peptides that promote inflammation resolution 50, and targeting peptides such as LyP-1 that facilitate plaque-specific delivery 51. These approaches primarily act by modulating lipid metabolism, inflammatory signaling, or vascular targeting. In contrast, our peptide directly targets the IP_3_R-GRP75 interaction at MAMs, thereby regulating ER-mitochondrial Ca²⁺ transfer and autophagy. This distinction highlights a complementary therapeutic strategy that operates at the inter-organelle level. From a therapeutic perspective, peptide-based modulation of MAM-resident PPIs offers potential advantages, including specificity, tunability, and compatibility with complex intracellular signaling hubs 21. This concept is supported by emerging evidence that functional specificity within protein complexes can be achieved through selective modulation of interaction states rather than complete disruption of individual proteins 52, 53. The *in vivo* efficacy observed in a Western diet-induced atherosclerosis model supports the translational relevance of this approach and suggests potential applicability to disorders associated with lipid stress and impaired autophagy. In addition, the ability to quantify MAM architecture and IP_3_R-associated interactions *in vivo* suggests that organelle contact remodeling may serve as a pharmacodynamic indicator of therapeutic response.

We acknowledge that a direct biodistribution analysis would further strengthen this study, and this will be an important subject of our future investigation. Although we did not directly examine the *in vivo* biodistribution of the peptide, the inclusion of the Hph-1 protein transduction domain supports its potential for systemic delivery. Previous studies have shown that Hph-1-conjugated proteins can be delivered effectively to multiple tissues after systemic administration and exert biological effects *in vivo* 30-32. Consistent with this, we observed significant changes in atherosclerotic burden and MAM architecture in cardiovascular tissues, indicating that the peptide reaches disease-relevant sites. Further studies on tissue distribution and cell type-specific targeting will be important to better understand its *in vivo* delivery profile. In this study, the peptide was administered at a dose chosen to provide adequate systemic exposure for evaluating therapeutic effect in a preclinical atherosclerosis model. Therefore, the dosing regimen should be interpreted in the context of a proof-of-concept study. Importantly, no apparent signs of systemic toxicity were observed during treatment, and treatment improved circulating lipid profiles and reduced atherosclerotic burden. Nevertheless, further optimization of peptide stability, pharmacokinetics, and dosing will be necessary to improve translational feasibility. Future efforts to improve peptide stability and tissue distribution, as well as to expand this strategy to additional MAM interactions and disease models, may further advance the development of precision therapies targeting organelle contact sites in metabolic and cardiovascular diseases.

## Supplementary Material

Supplementary figures.

## Data Availability

All original data and materials for this study can be obtained from the corresponding author upon reasonable request.

## Abbreviations

AMPK: AMP-activated protein kinase
ApoE: apolipoprotein E
ATP: adenosine triphosphate
BODIPY: boron-dipyrromethene
Ca^2+^: calcium ion
CETSA: cellular thermal shift assay
CQ: chloroquine
CPP: cell-penetrating peptide
DQ-BSA: dye-quenched bovine serum albumin
ER: endoplasmic reticulum
GRP75: glucose-regulated protein 75
HUVECs: human umbilical vein endothelial cells
IP: immunoprecipitation
IP_3_R: inositol 1,4,5-trisphosphate receptor
K_d_: dissociation constant
LC3: microtubule-associated protein 1 light chain 3
MAM: mitochondria-associated ER membrane
MST: microscale thermophoresis
NT: non-treated control
oxLDL: oxidized low-density lipoprotein
PLA: proximity ligation assay
PM: plasma membrane
PPI: protein-protein interaction
RAW264.7: murine macrophage cell line
SBD: substrate-binding domain
SEM: standard error of the mean
siRNA: small interfering RNA
SPLICS: split-GFP-based contact site sensor
TFEB: transcription factor EB
TMD: transmembrane domain
VDAC1: voltage-dependent anion channel 1
