# Supplementary material for: A MAM-targeting therapeutic peptide restores autophagy homeostasis and ameliorates atherosclerosis
Source: Theranostics. 2026 Apr 16;16(11):6113–31. doi: 10.7150/thno.132357 (PMC13142235; doi:10.7150/thno.132357)
Supplement: Supplementary file 1 — Supplementary figures. [file thnov16p6113s1.pdf]

Supplementary Materials

A MAM-targeting therapeutic peptide restores autophagy homeostasis and ameliorates atherosclerosis

Jungmin Ha[1], Minjeong Ko[1], Yong-Beom Lim[2], and Ho Jeong Kwon[1,*]

[1]*Chemical Genomics Leader Research Laboratory, Department of Biotechnology, College of Life Science and Biotechnology, Yonsei University, 50 Yonsei-ro, Seodaemun-gu, Seoul 03722, Republic of Korea.*

[2]*Department of Materials Science and Engineering, Yonsei University, 50 Yonsei-ro, Seodaemun-gu, Seoul 03722, Republic of Korea.*

*Corresponding Author: Ho Jeong Kwon, kwonhj@yonsei.ac.kr

Tel: +82-2-2123-5883, Fax: +82-2-362-7265

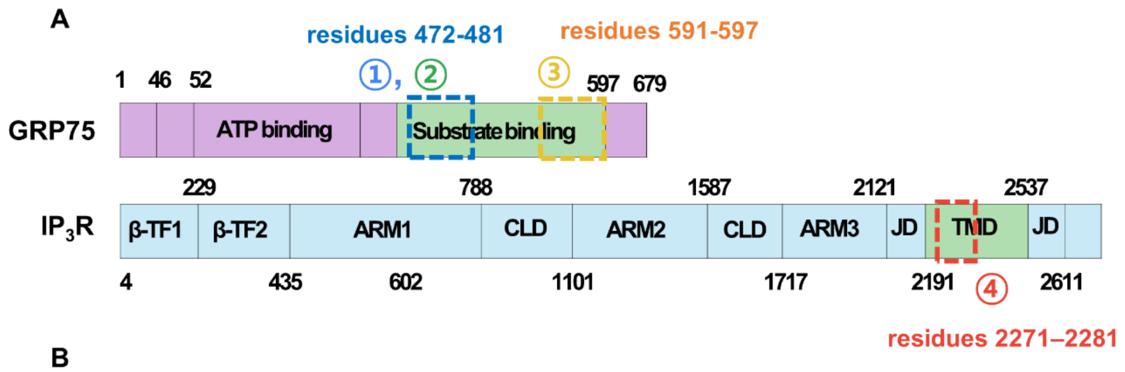

B

Peptide #	M.W. (Da)	pI	Length (mer)	CPP(Hph-1)	Sequence
①	2450	12.0	21	YARVRRRGPRR	FSTAADGQTQ
②	2621	12.0	24	YARVRRRGPRR	GGG FSTAADGQTQ
③	2278	11.3	18	YARVRRRGPRR	DTETKME
④	2664	11.9	22	YARVRRRGPRR	RSIYYLGIGPT
④ mutant	2480	12.4	22	YARVRRRGPRR	RSIAALGIGPT

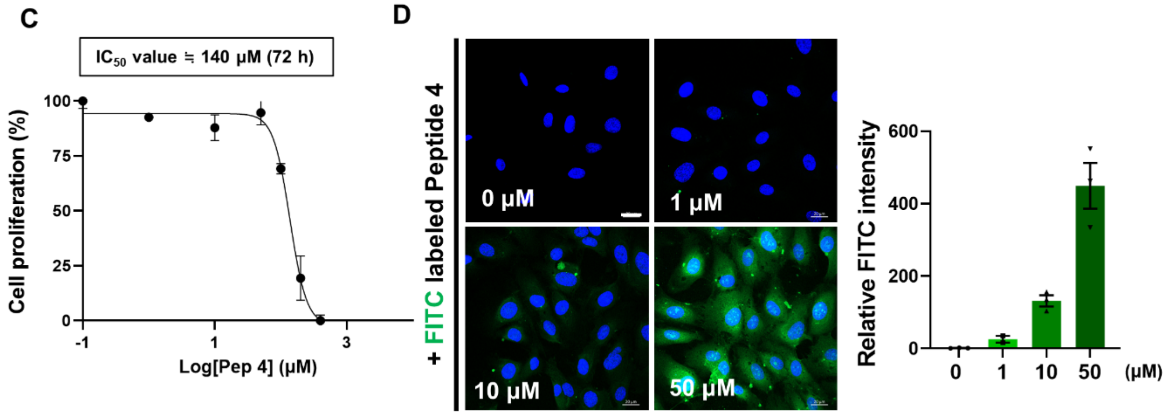

Figure S1. Peptide design strategy based on GRP75-IP$_3$R domains and validation of cell permeability and cytotoxicity

(A) Schematic illustration of the domain organization of GRP75 and IP$_3$R used for peptide design. The substrate-binding domain (SBD) of GRP75 and the transmembrane domain (TMD) of IP$_3$R are indicated. Peptide sequences derived from the GRP75 SBD (Peptides 1-3) and the IP$_3$R TMD (Peptide 4) are shown with their corresponding residue positions. (B) Summary of the physicochemical properties and sequences of the designed peptides. Molecular weight (M.W.), isoelectric point (pI), and peptide length are shown for each candidate peptide. All peptides were conjugated to the Hph-1 cell-penetrating peptide (CPP) at the N-terminus. A mutant control peptide was generated by alanine substitution of key residues within the predicted interaction interface. (C) Cell viability of human umbilical vein endothelial cells (HUVECs) following 72 h treatment with Peptide 4 at the indicated concentrations (0-400 μM),

assessed by MTT assay. Data are presented as percentages relative to untreated controls (mean ± SEM, n = 3). (D) Representative fluorescence images showing intracellular uptake of FITC-labeled Peptide 4 in HUVECs following 1 h treatment at the indicated concentrations (0, 1, 10, and 50 μM). Nuclei were stained with DAPI (blue). The right panel shows quantification of relative intracellular FITC fluorescence intensity. Scale bar, 20 μm.

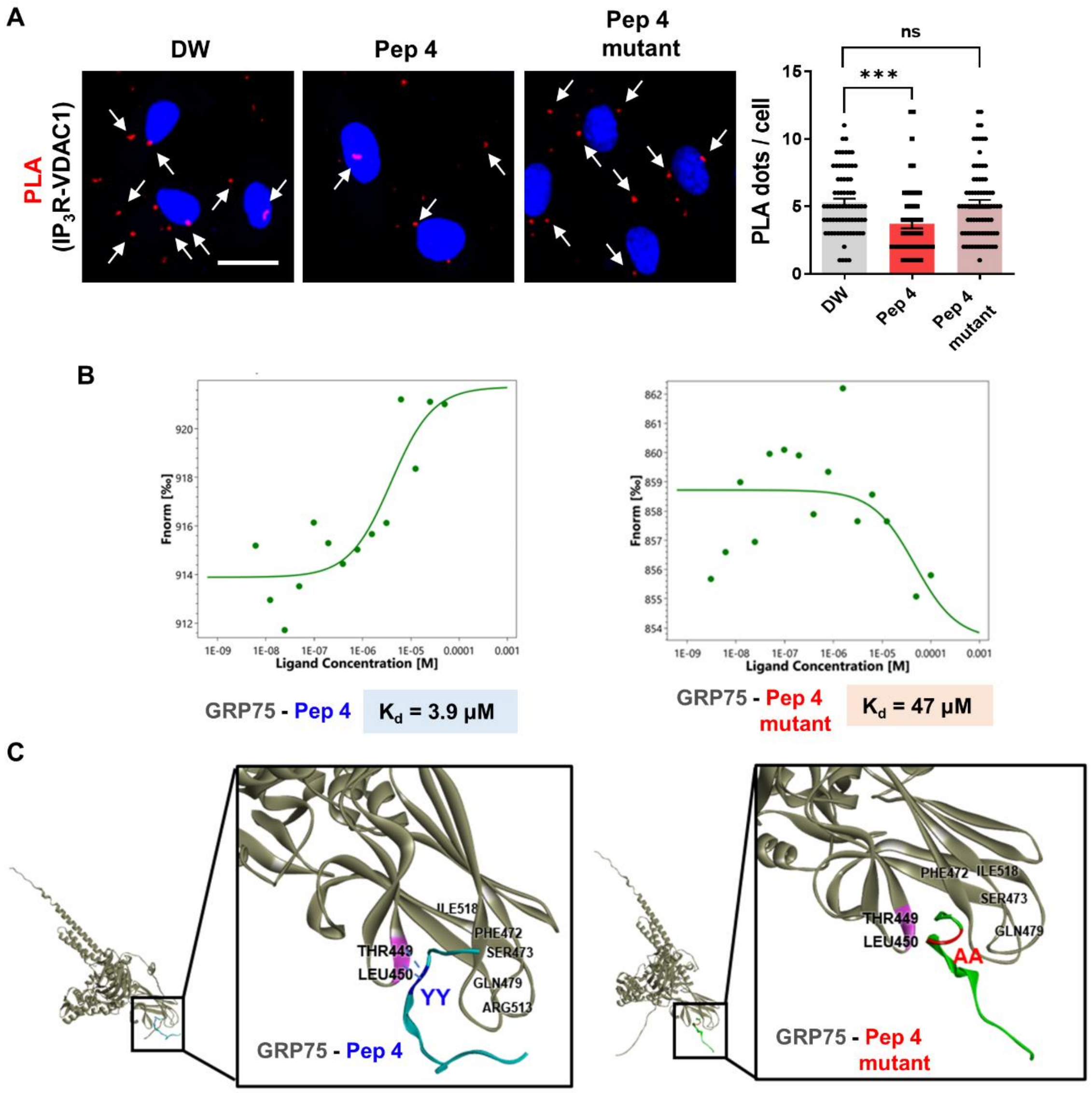

Figure S2. Sequence-specific disruption of IP$_3$R-GRP75 interaction and validation of direct binding by Peptide 4.

(A) Proximity ligation assay (PLA) analysis of mitochondria-ER interactions in HUVECs treated with Peptide 4 or its mutant control peptide (Pep 4 mutant). The mutant peptide was generated by substituting two tyrosine residues in Peptide 4 with alanine (YY→AA). Representative images and quantification of PLA puncta are shown. White arrows indicate representative PLA puncta. Scale bar, 20 µm. (B) Microscale thermophoresis (MST) analysis of the binding affinity between GRP75 and Peptide 4 or its mutant peptide. Peptide 4 exhibited a binding affinity (K_d) of 3.9 µM, whereas the mutant peptide showed reduced binding (K_d = 47 µM). (C) Structural models of GRP75 in complex with Peptide 4 (Pep 4) or the mutant peptide (Pep 4 mutant) were generated using AlphaFold-multimer, illustrating the predicted binding configurations. Data are presented as mean ± SEM. Statistical significance was defined as ***P < 0.001; ns, not significant.

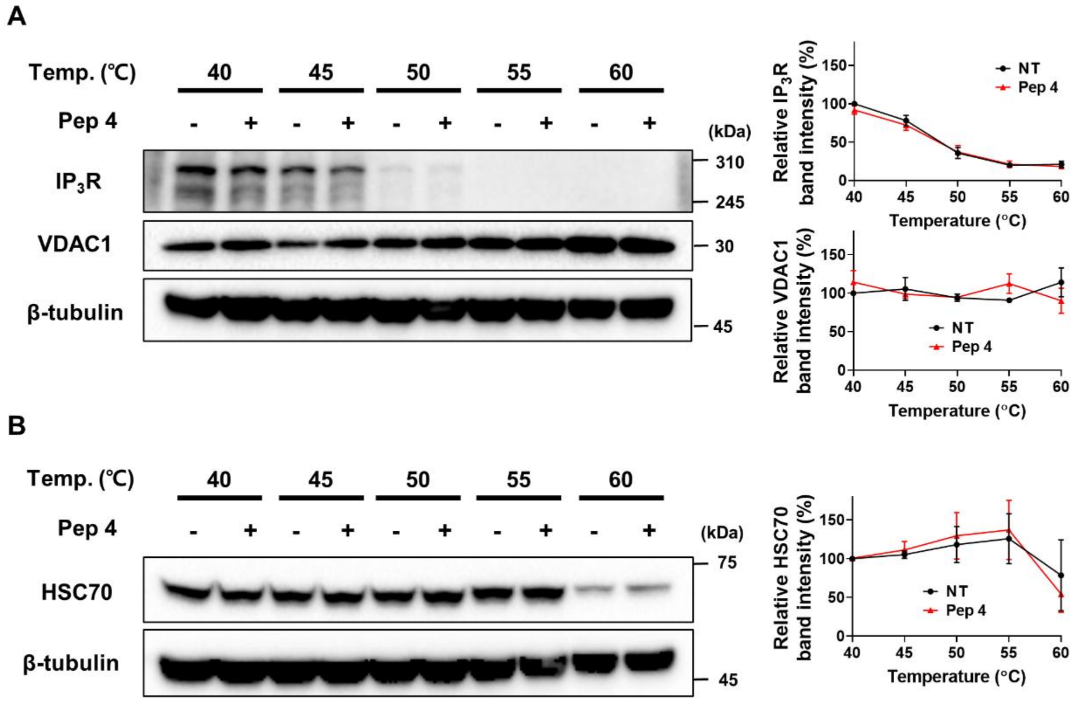

Figure S3. Selective binding of Peptide 4 to GRP75 assessed by CETSA.

(A) Cellular thermal shift assay (CETSA) analysis of IP_3R and VDAC1 in HUVECs treated with Peptide 4 for 1 h, followed by heating at the indicated temperatures (40-60 °C). Representative immunoblots and corresponding quantification of soluble protein levels are shown. (B) CETSA analysis of HSC70 under the same experimental conditions. Data are presented as mean ± SEM.

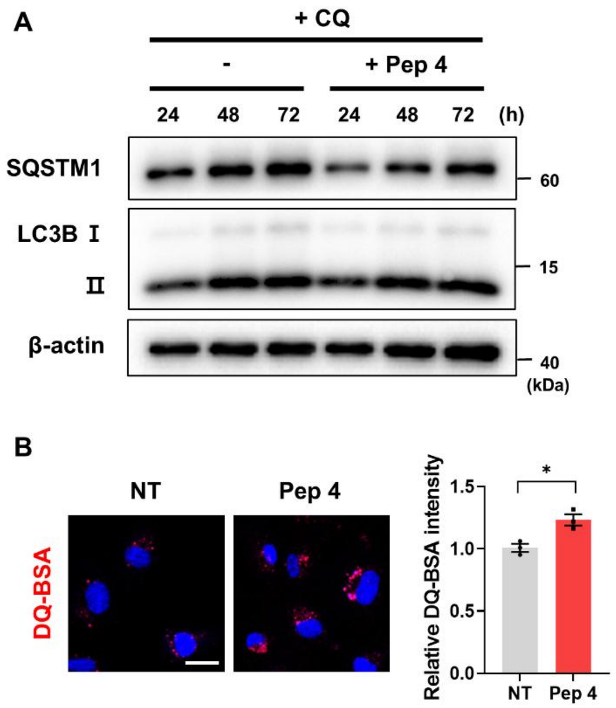

Figure S4. Peptide 4 promotes autophagic flux and lysosomal proteolytic activity in HUVECs.

Human umbilical vein endothelial cells (HUVECs) were treated with Peptide 4 (100 μM) or non-treated (-) for the indicated durations prior to analysis. (A) Immunoblot analysis of LC3B-I/LC3B-II conversion and SQSTM1/p62 levels in cells treated with Peptide 4 in the presence or absence of chloroquine (CQ; 10 μM), a lysosomal inhibitor, to assess autophagic flux. (B) Lysosomal proteolytic activity assessed by DQ-BSA fluorescence in control and Peptide 4-treated cells (100 μM, 12 h). Scale bar, 20 μm. Data are presented as mean ± SEM. Statistical significance was defined as $*P < 0.05$.

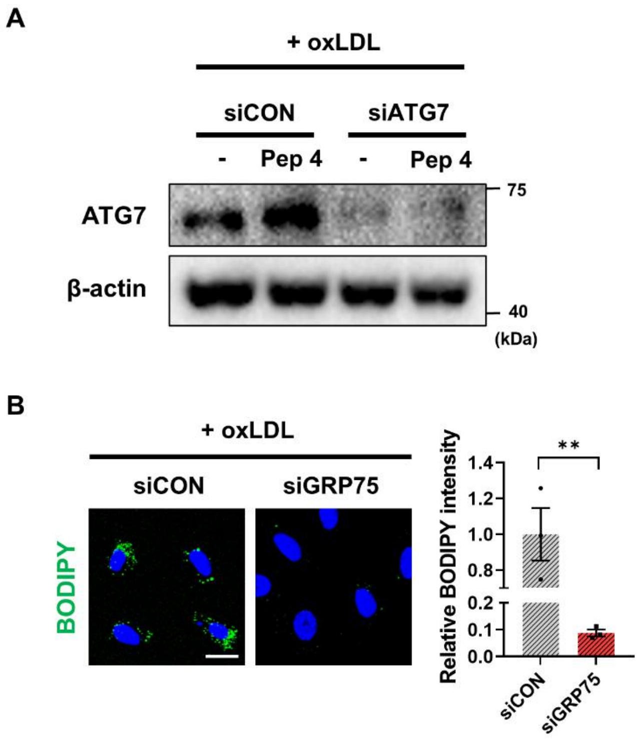

Figure S5. Effects of ATG7 or GRP75 knockdown on lipid accumulation in HUVECs.

(A) Immunoblot analysis was performed to confirm the knockdown of ATG7 following 24 h of siRNA treatment in HUVECs. (B) HUVECs were subjected to GRP75 knockdown for 24 h, followed by treatment with oxidized LDL (oxLDL; 50 µg/mL) for 48 h. Lipid accumulation was assessed by BODIPY staining. Representative fluorescence images and corresponding quantification of relative BODIPY intensity are shown. Scale bar, 20 µm. Data are presented as mean ± SEM. Statistical significance was defined as $^{**}P < 0.01$.
